# The Effects of Urban Neighborhood Environmental Evaluation and Health Service Facilities on Residents’ Self-Rated Physical and Mental Health: A Comparative and Empirical Survey

**DOI:** 10.3390/ijerph19084501

**Published:** 2022-04-08

**Authors:** Jiangjun Wan, Yutong Zhao, Yun Chen, Yanlan Wang, Yi Su, Xueqian Song, Shaoyao Zhang, Chengyan Zhang, Wei Zhu, Jinxiu Yang

**Affiliations:** 1 School of Architecture and Urban-Rural Planning, Sichuan Agricultural University, Chengdu 611830, China; wanjiangjun@sicau.edu.cn (J.W.); 201608383@stu.sicau.edu.cn (Y.Z.); chenyun@stu.sicau.edu.cn (Y.C.); wangyanlan@stu.sicau.edu.cn (Y.W.); 41358@sicau.edu.cn (C.Z.); zhuwei@sicau.edu.cn (W.Z.); 2 Rural Development Research Institute, Sichuan Academy of Social Science, Chengdu 610041, China; 2020425034@stu.sicau.edu.cn; 3 School of Management, Chengdu University of Information Technology, Chengdu 610225, China; sxq@cuit.edu.cn; 4 College of Geography and Resources Science, Sichuan Normal University, Chengdu 610101, China; zhangsyxs@sicnu.edu.cn; 5 School of Economics, Sichuan Agricultural University, Chengdu 610101, China

**Keywords:** self-rated health, neighborhood environment, health service facilities, Chengdu

## Abstract

(1) Background: The neighborhood environment has been shown to be an essential factor affecting residents’ quality of life and health, but the relationship between the characteristics of health service facilities and health levels is rarely known. (2) Methods: This study used a representative sample (*n* = 591, 303 women; 288 men, age 18–85 years, lived in Chengdu for an extensive time) of residents living in Chengdu City, China, and took spatial point data and empirical research data to construct an ordered logistic regression model. We contrastively analyzed the influence of different variables in the neighborhood environment and health service facilities on self-rated physical health (SRPH) and self-rated mental health (SRMH). (3) Results: The frequency of use and accessibility of multiple facilities in the health service facilities were significantly associated with self-rated health (SRH). Significant differences occurred between residents’ perceived accessibility and actual accessibility of facilities in SRH. Comparing the results of SRPH and SRMH revealed that the influencing factors that affect the two vary. The factors that significantly affect SRMH include neighborhood physical environment evaluation; social environmental evaluation; the frequency of use of the parks and squares, and sports zones; and the accessibility of parks and squares, specialized hospitals, community hospitals, and pharmacies. However, the factors that significantly affect SRPH include the frequency of use of sports venues, general hospitals, and pharmacies and the accessibility of general hospitals. The social environment of the neighborhood is also a non-negligible part, and its interaction with the physical environment of the neighborhood affects the outcome of SRH. (4) Conclusions: Neighborhood environmental characteristics and the layout of health service facilities have significant differential effects on people’s physical and psychological health, and this information is of great value in promoting healthy city development and improving the quality of life of urban populations around the world.

## 1. Introduction

The relationship between the urban environment and health has always been a research hotspot [1]. The health benefits of the urban environment depend on the management of key environmental and facilities that affect health. Among these determinants, the neighborhood environment and health service facilities are closely related to people’s lives, and they have always attracted the attention of scholars. Neighborhood environments and health service facilities will affect the health of residents [2], but the difference in the physical and mental health of residents is currently relatively vague. To date, the main research perspective has been to analyze the effects on physical and mental health from a single environment or facility alone [3]. However, in order to better understand the complexity of urban neighborhood environment and health service facility factors, the demand for comprehensive studies integrating multiple factors and multi-level frameworks has increased in recent years. We analyze the key differential factors affecting self-rated physical health (SRPH) and self-rated mental health (SRMH) from a comparative research perspective (differences in the effects of neighborhood environment and health service facilities on SRPH and SRMH), combining subjective surveys of residents and objective spatial measures. In the paragraphs to follow, the existing literature is discussed and interpreted under the different components of this objective.

### 1.1. Literature Review

#### 1.1.1. Analysis of SRH and Influencing Factors

Self-rated health (SRH) was first proposed in 1985 and has increasingly become an important indicator of health research. The characteristics of SRH are the comprehensive multidimensional assessment of the interviewees’ own health, including the physical, psychosocial and social dimensions of health, as well as the current health status and future health expectations. Some studies confirmed that objective and SRH are consistent and that SRH levels can, to some extent, reflect the overall actual health state of inhabitants. As an indicator of overall health, SRH can predict future health better than other more objective indicators. SRH is often measured using the Likert scale, in which respondents select sequential options such as “very good, good, fair, poor, very poor.” The outcomes are compared to either a control group or a self-perceived ideal state of health.

Relevant studies have shown that age, gender, education, work satisfaction, healthy living habits, income, economic and social status, and neighborhood factors significantly impact SRH [4,5]. Some scholars have studied the impact of psychosocial factors on SRH and pointed out that psychosocial factors are key determinants of the level of SRMH and that poor SRH may be an outward manifestation of negative psychosocial conditions (e.g., dissociation, lost life events, depression, and work stress) [6]. Some researchers have also examined the differences in SRH levels among different groups and the factors that influence them by focusing on persons from different areas, ages, genders, and income levels [7,8].

Scholars have recently begun to deepen their research focus on the importance of SRMH [9]. In most studies, SRMH is measured using a series of basic questions that ask respondents to rate their overall health, mental health, and mood on a scale of poor to excellent and then determine the SRHM according to their responses. Thus, SRMH, similar to global SRH measurements, may include dimensions not observed in objective health, such as severity, illness persistence, and functional limits. In fact, the consistency of SRMH and actual mental health problem assessments is related to the severity of the impairment as well as the degree of impaired functioning in the majority of research [10,11]. The preceding results have taken SRH as a scientific health measuring approach, thereby demonstrating that investigating its affecting variables and formation mechanism is crucial to enhancing population health.

#### 1.1.2. Urban Neighborhood Environment and Health

More than half of the world’s population now lives in cities as a result of accelerating global urbanization. The WHO has emphasized the importance of paying attention to an urban environment’s friendliness to people, aiming to help people stay healthy and active [12]. An urban environment is mainly divided into physical and service environments, and numerous studies focus on the respective impacts of those environments on the health of residents. Land, air, water, and vegetation constitute a city’s physical environment. A city’s service environment is represented in the quality of facilities and service levels, such as public service facilities and housing supplies. Within the urban environment, the physical environment is dominated by the built environment, and the social environment includes aspects of social organization, such as civic involvement, environmental policies and regulations, and community investment [13,14].

Urban environments offer many advantages for health (e.g., access to employment opportunities and proximity to health care services and health facilities) but also present various challenges, including increased exposure to health hazards (e.g., air pollutants, occupational health risks, traffic, and crime) [15,16]. Furthermore, access to the benefits and risks associated with an urban environment is unequally distributed, as are the health implications [17,18]. Given the uncertainty of the geographical background, the objectively measured environmental variables may not reflect the actual environmental exposure of the residents, making it difficult to fully capture the health effects of the environment [19]. Therefore, neighborhood environment measures in line with residents’ perceptions and interactions have become an important avenue of research by assessing the local social environment and the physical environment in which urban residents live and interact and analyzing the effects of these factors on health outcomes [20].

In health research, the neighborhood (or community) environment refers to the immediate environment in which residents live and is assumed to have the physical and social elements associated with health [21]. Investigations on the impact of neighborhood environment on health in Western countries began in the 1990s when American sociologist Wilson created the notion of the neighborhood effect and discovered that neighborhood characteristics had a negative impact on residents’ attitudes and social actions in a study of slums. The dimensions of neighborhood characteristics commonly studied can be divided into socio-economic (income and schooling and economic inequality), physical (landscape, pollution, and service infrastructure), and psychosocial (social participation, respect, and social inclusion) components [22,23,24]. Poor environmental conditions often have a negative impact on health, thereby increasing the likelihood of residents believing that they are sick. Numerous studies have shown that better neighborhood environments can promote physical and mental health [25]. Simultaneously, several articles have focused on physical activity as a feature of the neighborhood environment, as well as other routes linked to health consequences [26]. The existing literature has confirmed a relationship between the high evaluation of neighborhood buildings and social environment perception and the possibility of reporting good health. Related studies have also indicated that appearance characteristics, congestion, air pollution, noise, living conditions, transportation convenience, and open space are all linked to health-related key physical environmental factors [27,28]. Similarly, psychosocial factors are not easily overlooked, with some studies confirming that social engagement, respect, and social inclusion are associated with higher odds of reporting good health and that higher rates of social engagement, social support, and interaction with neighbors positively impact SRH [29]. Clearly, neighborhood environmental characteristics play a key role in SRH, but few studies have focused on the effects of the combined physical and social environment of a neighborhood on SHR, and scarce research has compared and analyzed the differential effects of the neighborhood environment on SRPH and SRMH.

#### 1.1.3. Health Service Facilities and SRH

The investigation of the relationship between health service facility factors and SRH is more limited to investigations of the influence of neighborhood environment on SRH, with a current emphasis on the particular influence of health care facility use and satisfaction and accessibility of parkland. These studies establish that the frequency of medical facility usage is negatively correlated with SRH, satisfaction with residential and community green spaces is positively correlated with residents’ SRH outcomes, and accessibility of green park spaces is also positively correlated with SRH [30,31,32]. However, a hypothesis suggests that the association between public green space and SRH varies by vegetation type and that not all types of vegetation are beneficial in improving SRH [33]. In general, studies on one type of health service facilities and SRH outnumber those that synthesize the effects of multiple types of health service facilities on SRH, and the latter must be explored further.

### 1.2. Theoretical Framework

**Figure 1:**
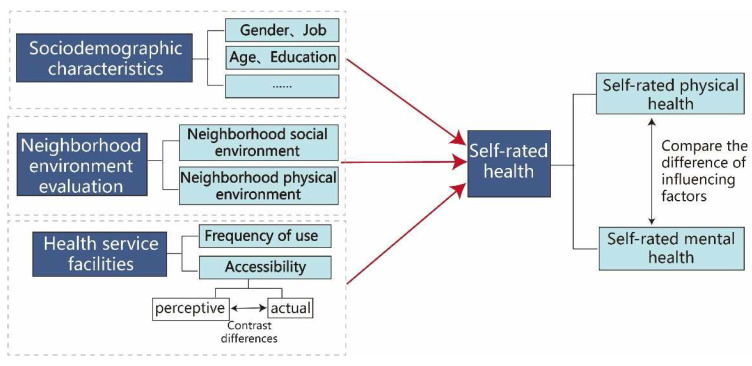
Research framework.

The effect of neighborhood health facilities (health and physical activity facilities) on residents’ SRH has received minimal attention in the literature, and whether the effect of neighborhood environmental characteristics on SRH and SRMH varies remains unclear. On this basis, this article takes the residents of Chengdu’s main urban area as its research object to explore the relationship between a neighborhood’s physical and social environment and SRH. This work addresses four issues: 1. identifying the key influences of residents’ perceived neighborhood physical and social environments and SRH; 2. ascertaining whether differences occur in the effects of neighborhood environmental characteristics on SRPH and SRMH; 3. clarifying whether residents’ neighborhood health facility use habits affect SRH; and 4. detecting differences between residents’ perceived and actual health service facilities and improvement measures (Figure 1).

## 2. Methods

### 2.1. Study Area

**Figure 2:**
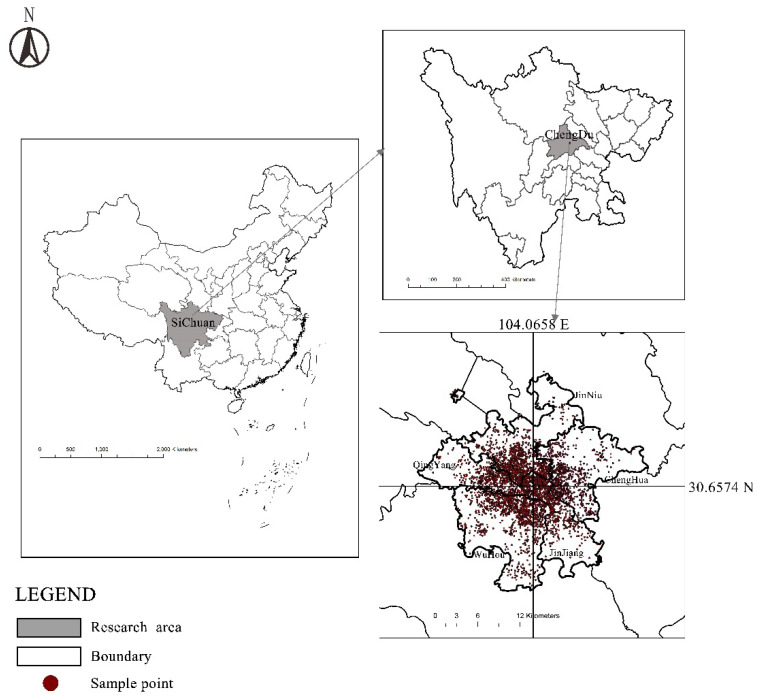
Location of the study area.

The study area of this paper is the central urban area of Chengdu City, Sichuan Province, with longitude and latitude of 104:04 E and 30:39 N, respectively (Figure 2). The total land area is approximately 424.06 square kilometers. In 2019, the total population of permanent residents was 5.677 million, with a male to female ratio of 1.052. By the end of 2016, the population density in the urban core of Chengdu reached 10,300 people per square kilometer. With Chengdu’s rapid development, problems such as traffic congestion, serious environmental issues, a lack of public service facilities, and a lack of public space have recently emerged in the central urban area, and all of these factors have a significant impact on residents’ health. As a result, the central urban area of Chengdu is chosen as the case area of this study [34,35].

### 2.2. Data Collection

The study collected two types of data:

The first category is the field survey data collected by issuing questionnaires and undertaking in-depth case interviews. During the investigation (4 July 2020–27 September 2020), we conducted random interviews with residents of different neighborhoods in Chengdu’s central urban area from 9:00 a.m.–10:00 p.m. to collect data from various time periods. First, a pre-survey was carried out. After collecting the effective pre-survey questionnaires, reliability and validity tests were performed on the pre-survey questionnaire data results, and the final questionnaire was revised, developed, and distributed. Before distributing the questionnaire in this study, we needed to determine whether the interviewees lived in Chengdu’s downtown area for an extended time (more than half a year) to ensure that all of the interviewees are residents who have lived in the study area for a long time. A total of 674 questionnaires were issued. In total, 591 of these were valid questionnaires, accounting for 87.7% of the distributed questionnaires.

Structured questionnaires consisting of three parts were used in the study. The first part investigated residents’ evaluation of the neighborhood physical environment and the neighborhood social environment, as well as the usage frequency (average monthly frequency) of various health service facilities in the neighborhood. The second part collected the SRH status from residents. At the same time, considering the influence of different individuals on SRH, we classified individual factors, so the third part involves the personal characteristics of respondents, including multiple independent individual variables such as gender, age, education level, occupational status, income, specific address, and residential types. In this work, the evaluation level is set as 5. In order to facilitate quantitative analysis, the numerical values are assigned as follows: 5 points—excellent, 4 points—good, 3 points—poor, 2 points—poor, and 1 point—very poor.

The second category is geospatial data, which mainly include the point of interest (POI) data of various health service facilities and residential areas, and road traffic network. We used Python to determine the POI data of health service facilities and residential areas in the downtown area of Chengdu in 2019 on an AutoNavi map. POI is the point-specific data of spatial entities that are directly tied to daily lives and contains exact geographic and attribute information, such as latitude and longitude, name, address, type, and label [36]. They provide accurate location and detailed category information of business places, living services, and public places, with the advantages of extensive and free access.

In the analysis of the impact of perceived and actual accessibility of health service facilities on SRH, subjects that do not disclose their residential area’s specific address in the questionnaire were eliminated from the evaluation. A total of 308 samples were removed, thereby resulting in 283 remaining samples. The chi-square test was used to compare the impact significance between the actual and perceived accessibilities of types of health service facilities.

### 2.3. Variables Analyzed

#### 2.3.1. Dependent Variable

The dependent variable is the residents’ SRH, including their SRPH and SRMH (Table S1).

(1) SRPH

Respondents were asked to rate their overall health on a five-point Likert scale, with the question: “Overall, how do you rate your health compared to your peers?” In the scale, 1 = Very Poor, 2 = Poor, 3 = General, 4 = Good, and 5 = Very Good.

(2) SRMH

We measured the mental health status of residents by using the Warwick-Edinburgh Positive Mental Health Scale (WEMWBS).

The WEMWBS is a population-based scale designed to assess people’s positive mental health. At present, WEMWBS is one of the few scales that use positive questions to monitor public mental health. Previous studies have shown that the scale has good reliability and validity and is effective for different populations [37]. This group includes adolescents, adults, elderly people, and individuals with mental illness in various countries [37,38,39]. WEMWBS scores are derived from responses to 14 positively expressed statements about subjective happiness and effective mental functioning [40].

We asked respondents to select the statement that best described the past two weeks and use the following five-point Likert scale (not often, rarely, sometimes, often, and always). With every index score ranging from 1 to 5, the sum of the rankings is from 14 (worst mental health) to 70 (best mental health). High WEMWBS scores mean high levels of mental health. In comparing the impact of the differences of the same factors on the SRPH and SRMH of respondents, the average value of the mental health scale score is taken, and the score is divided into five grades (0–1 = poor, 1–2 = average, 2–3 = good, 3–4 = very good, and 4–5 = excellent) [40].

#### 2.3.2. Independent Variables Related to Neighborhood Environment

##### Individual Characteristics

We control for individual characteristics, including age, gender, marital status, employment status, education level, personal annual income, and community quality, that might significantly moderate residents’ SRH outcomes. Community quality is divided into ordinary commercial housing or affordable housing district, high-end commercial housing or senior residential district or villa district, unit community or school dormitory, unreconstructed old city, shantytown, and rural residential community. To analyze and test the differences in residents’ SRH, we divided the residential types into four categories: low-grade housing (includes shantytowns, unreconstructed old city housing, and rural residential housing); middle-grade housing (includes common commercial housing), high-grade housing (includes villas and high-grade commercial housing), and fourth-grade housing (includes units and school dormitories).

##### Neighborhood Environmental Evaluation

This study only considered neighborhood environment factors on SRH, so for the time being, poor personal habits and major diseases are not taken into account. Studies have shown that residents’ evaluation of the environment significantly impacts residents’ SRH more than the objective environment. Therefore, we choose neighborhood environmental evaluation as the critical variable for the study [41]. Neighborhood environmental evaluation includes two dimensions: neighborhood physical environment evaluation and neighborhood social environment evaluation. Physical environment characteristics of neighborhoods associated with health include the natural environment and public service facilities (sports facilities and medical and health facilities). In this study, neighborhood physical environment evaluation involves air quality, ecological water quality (water quality of surrounding rivers and lakes), urban greening, garbage disposal (community garbage disposal and sanitation of public places), health service facilities (sports facilities: sports venues and sports zones, parks and squares; medical and health facilities: general hospitals, specialized hospitals, community hospitals, clinics, and pharmacies). The evaluation of health service facilities is investigated separately from convenience evaluation and service evaluation. The average value of the two is taken during calculation. Existing studies have shown that the characteristics of the neighborhood social environment related to health mainly consist of community safety [42], neighborhood interaction, social network [43], etc. Therefore, neighborhood social environment evaluation in the study includes three aspects: neighborhood relationship, social interaction environment, and urban public security environment.

Factors such as accessibility of health service facilities and the frequency of residents’ use of health service facilities are also highlighted as these characteristics may play an important role in influencing residents’ SRH outcomes. The perceived walking time from the residence to the facility determines the accessibility of health service facilities.

### 2.4. Statistical Analysis

First, we used descriptive statistical methods to ascertain the basic characteristics and the basic situation of the respondents’ SRH. We constructed a logistic regression model and explored the effects of all types of variables on residents’ SRPH and SRMH. Before the creation of the model, an ordered logistic regression analysis was performed on the demographic variables and dependent variables to eliminate demographic variables that failed to show statistical significance at the 95% confidence level.

Then, demographic and sociological characteristics, neighborhood environmental variables, socio-economic variables, and health service facility usage habits were included in the model. The results of SRPH and SRMH were compared and analyzed to explore the difference in relation to the influence of the same factors on SRPH and SRMH.

Finally, the chi-square test was applied to evaluate the impact of the actual accessibility and perceived accessibility of health facilities on SRH. The questionnaire ascertained the residence locations of respondents and the residents’ perceived accessibility to health service facilities. The perceived accessibility of health service facilities is the respondents’ answer to the question “the time it takes to walk to the nearest place from home,” which is categorized into less than 5 min, 5–10 min, 11–15 min, 16–20 min, more than 20 min, and unclear. The actual accessibility of health service facilities was determined according to the address provided by the interviewees. A network data set was created in Geographical Information Systems (Arc-GIS) to generate the corresponding range and calculate the actual walking time of 5, 10, 15 or 20 min from their residence to the nearest facility. THE SPSS (version 25.0; Statistical Product and Service Solutions, Chicago, IL, USA) software was utilized for the statistical analysis of the entire data set.

## 3. Results

### 3.1. Descriptive Statistical Results

#### 3.1.1. SRH Outcomes in Populations with Different Socio-Demographic Characteristics

Descriptive statistical results of variables (Table 1) revealed significant differences in the SRPH and SRMH levels of residents with different genders and marital statuses. Men have a higher average SRMH and SRPH score than women.

Additionally, significant differences occurred in the SRMH of residents in different age groups and with different annual incomes. The average level of residents’ SRMH increases with age, and the elderly have the highest SRMH score (4.14). People with an annual income exceeding 100,000 RMB (3.85) have the highest SRMH, followed by those with an annual income under 5000 RMB (3.77). Residents with an annual income in the middle level (3.66) have the lowest SRMH.

#### 3.1.2. Analysis of Neighborhood Environmental Evaluation

The scores for neighborhood physical and social environment evaluation were collected by questionnaire, and the average values were calculated. The factors for neighborhood physical environment evaluation ranked from high to low are as follows urban greening > air quality > waste disposal > ecological water quality > medical sanitation > sports facilities, neighborhood social environment evaluation from high to low in turn as follows: urban public security environment > social environment > community neighborhood relations (Table 2).

With averages of 3.69 and 3.39, respectively, neighborhood social environment evaluation is significantly higher than neighborhood physical environment evaluation, thereby indicating that residents are relatively satisfied with the neighborhood’s physical environment and social environment in Chengdu.

### 3.2. Associations between Neighborhood Environment Characteristics and SRH Outcomes

To explore the correlation between neighborhood environment characteristics and SRPH and SRHM, we include residents’ socio-demographic characteristics, frequency of use of health service facilities, and neighborhood environment characteristics as control variables in the model. In the data analysis, respondents with SRPH scores of 1–3 are classified as “poor”, 4 as “moderate”, and 5 as “good”. The same classification method was used for SRMH scores according to the average. In the multivariate ordered logistic regression model, we needed to ascertain whether the increasing gap of dependent variables is equal by employing a parallel line test. In the model of this study, when the SRPH and SRMH are divided into “poor,” “medium,” and “good” as dependent variables, the *p* value of the parallelism test of the model is larger than 0.05. Thus the evaluation intervals of “poor”, “medium”, and “good” are equidifferent.

**Figure 3:**
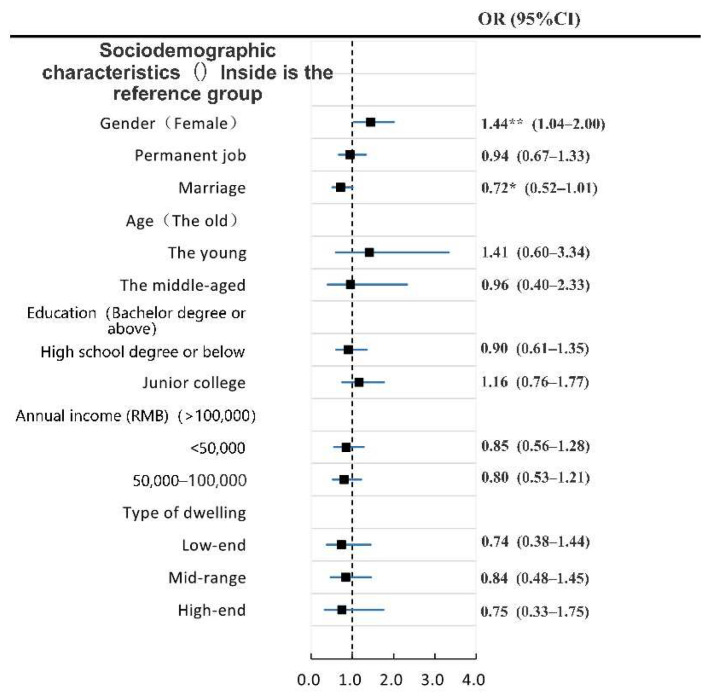
Univariate analysis of demographic variables, urban environmental evaluation and health facilities related variables and SRPH. Note: ** and * respectively represent *p* < 0.05, and *p* < 0.10. Variables (*n* = 591).

**Figure 4:**
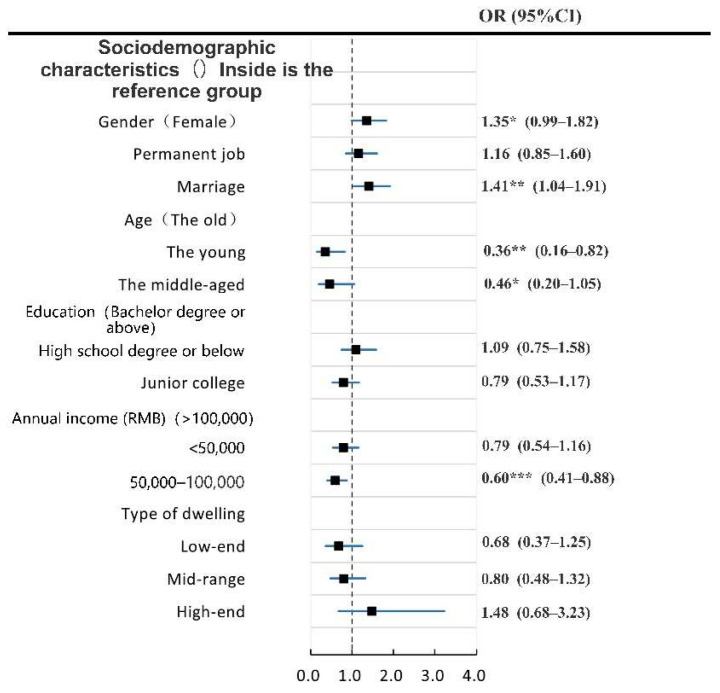
Univariate analysis of demographic variables, urban environmental evaluation and health facilities related variables and SRMH. Note: ***, ** and * respectively represent *p* < 0.01, *p* < 0.05, and *p* < 0.10. Variables (*n* = 591).

First, we analyzed the demographic variables and dependent variables in the model by ordered logistic regression (Figure 3 and Figure 4). A collinearity test was conducted for each independent variable to screen the factors. When SRPH and SRMH are taken as dependent variables, the variance inflation coefficient (VIF) of each factor is less than 5, thereby indicating that the collinearity problem between each factor is small and could be ignored. Some demographic variables that failed to show statistical significance at the 95% confidence level were excluded from the final model. In line with the analysis results, we deleted the variables “education level” and “occupation type” when mental health is the dependent variable. The other variables are retained in the final model. We deleted the variables “age”, “education level”, “occupation type”, “annual income”, and “housing type” when physical health is the dependent variable. The other variables were retained in the final model.

The model structure for SRPH and SRMH is the same, with Model 1 including only residents’ social demography variables, Models 2 and 3, respectively, include residents’ neighborhood physical environment evaluation and neighborhood social environment evaluation, Model 4 includes the residents’ frequency of health service facility use, and Model 5 includes the accessibility variables of health service facilities, which means all the variables are incorporated.

#### 3.2.1. Analysis of the Influencing Factors of SRPH

**Figure 5:**
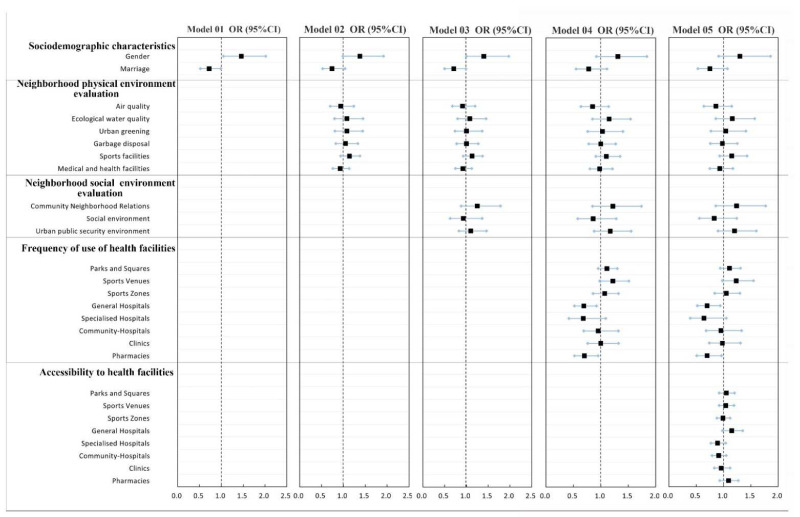
Ordered Logistic Regression Analysis of SRPH.

Model 1 demonstrates that gender and marital status are strongly related to SRPH. When the variables “neighborhood physical environment evaluation” (i.e., Model 2) and the variable “neighborhood social environment evaluation” (i.e., Model 3) are included, gender and marital status are still the factors affecting SRPH. In all models, neighborhood physical environment evaluation and neighborhood social environment evaluation show no significant correlation with SRPH (Figure 5).

Model 4 includes the residents’ frequency of health service usage and indicates that socio-demographic variables are not significant, but we retained them to adjust for other characteristics. At this point, the frequency of use of general hospitals is the variable with the greatest influence on the degree of SRPH (OR = 0.69). When the frequency of use of general hospitals increases by one unit, the probability of the SRPH level of residents decreases by 31%. The frequency of use of sports venues and pharmacies are also significant influencing factors. The frequency of use of sports venues is positively correlated with the SRPH level, and the frequency of use of pharmacies is the same as that of general hospitals. The higher the frequency of use, the worse the level of SRPH.

Figure 5 shows that the frequency of usage of sports venues, general hospitals, specialized hospitals and pharmacies, and the accessibility of general hospitals are strongly connected with SRPH when all variables are taken into account (Model 5). Compared with Model 4, Model 5 indicates that the frequency of use of specialized subject hospitals has become a significant influencing factor. When the frequency of use of sports venues and general hospitals increases by one unit, the probability of residents’ SRH increases. This outcome suggests that the variable “accessibility of health service facilities” in terms of the frequency of use plays a regulatory role in SRPH. The accessibility of general hospitals is positively correlated with SRPH (OR > 1), so higher accessibility of general hospitals may mean a higher SRPH level for residents.

#### 3.2.2. Influencing Factors of SRMH

**Figure 6:**
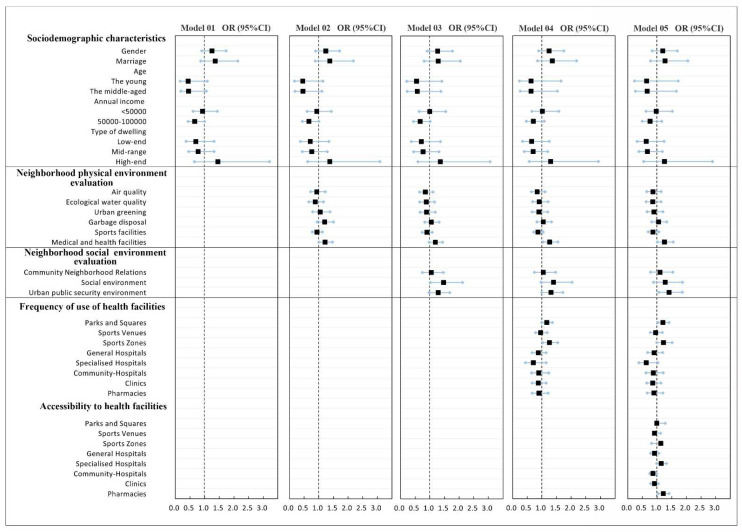
Ordered Logistic Regression Analysis of SRMH.

Figure 6 demonstrates that, in Models 1 and 2, residents’ age and income do not have a significant relationship with SRMH. Health facility evaluation is significantly correlated with SRPH in all models.

In Model 3, social environment evaluation has the greatest significant influence on the SRMH, followed by the urban public security environment. However, as the other variables are included in Model 4, the influence of social environment evaluation significantly decreases but still exerts the highest influence in the model. No significant correlation is found in Model 5.

Model 5 includes all variables and indicates that the frequency of use and accessibility of parks and squares are significantly positively correlated with SRMH, whereas the frequency of use of specialized hospitals and accessibility of community hospitals are negatively correlated with SRMH. Urban public security environment evaluation has the largest effect on residents’ SRMH. When urban public security environment evaluation increases by one unit, the residents’ SRMH level increases by 42%. The effect of other variables from the second strongest to the weakest effect is as follows: evaluation with medical and health facilities > the effect of frequency of use of sports zones > the effect of accessibility of pharmacies > the effect of frequency of use of parks and squares.

#### 3.2.3. Similarities and Differences of Factors Affecting SRMH and SRPH

The regression results of SRPH and mental health (Figure 5 and Figure 6) reveal that the factors affecting SRPH and mental health are significantly different. Factors within the two dimensions of neighborhood physical and social environment evaluation only have a significant impact on SRMH but have no significant impact on SRPH. Within the three dimensions of socio-demography, frequency of use, and accessibility of health facilities, the factors that significantly affect the SRPH and SRMH also vary. Among the factors, frequency of use of sports venues, general hospitals, and pharmacies and accessibility of general hospitals are the influencing factors of SRPH. The factors that significantly affect SRMH include the frequency of use of parks and squares and space zones and the accessibility of parks and squares, specialized hospitals, community hospitals, and pharmacies. Note that in Models 4 and 5, no significant correlation occurs between socio-demographic characteristics and SRPH and SRMH.

### 3.3. Actual and Perceived Accessibility of Health Facilities Have Different Effects on SRH

Table 3 shows that, in general, the impact on the actual accessibility of health service facilities is more significant than the perceived accessibility for SRPH. The influence of sports venues, sports zones, and a clinic presents a significant correlation between the actual accessibility of facilities to SRPH, but all these three facilities do not show a significant correlation between perceived accessibility to SRPH. The only difference is that the perceived accessibility of general hospitals has a significant impact on SRPH, but the actual accessibility does not. SRMH indicates just the opposite result to that of SRPH.

## 4. Discussion

### 4.1. Significant Differences Occur in the Effects of Socio-Demographic Characteristics on SRH

Social demographic traits represent an individual’s social, economic, educational, employment, marital status, and relationships and also influence residents’ neighborhood and community environment choices to some extent. Existing research established that socio-demographic variables have a mediating or moderating effect on residents’ SRH and constitute an essential component associated with individual health levels [44,45]. However, when other environmental variables are included in the logistic regression model, the influence of socio-demographic variables is considerably attenuated or entirely lost, thereby demonstrating that socio-demographic factors have a smaller impact on health than other aspects.

Women appear to be less likely than males to rank their health as good in most studies, on average, men’s SRMH and SRPH scores are higher than those of women, and our findings are consistent with those from previous studies [32,46]. However, when the sample consisted of female respondents, the SRPH was more likely to be good, according to the results of the SRPH model. This result could be linked to the long-held social beliefs that women spend the majority of their time close to home, are more susceptible to the influence of the neighborhood environment, and hence benefit more from their living environment [47].

A correlation was found between residents’ annual income and SRMH in the univariate analysis. Note that people with a middle-level yearly income had the lowest SRMH, an outcome that might be connected to China’s present middle-class anxiety. On the one hand, their anxiety stems from a sense of occupational and social crises. Despite having a larger income, they lack the tools of production, thereby putting them at a greater risk of unemployment. On the other hand, a sense of crisis emerges in relation to the family’s socio-economic status, as they worry that their children will be unable to move up further or at least inherit their socio-economic status [48]. Instead, those with the lowest incomes report higher levels of SRMH than those in the middle.

### 4.2. Neighborhood Environment, Health Service Facilities, and Other Factors Jointly Affect the Results of Residents’ SRH

#### 4.2.1. Future Planning Should Pay More Attention to the Creation of a Social Environment in the Community

Despite the fact that green space satisfaction and blue space are favorably connected to residents’ SRH and well-being, self-rated air pollution also has different effects on the SRH of groups from various socio-economic levels [49,50,51,52]. However, contrary to our prediction, the evaluation of urban air quality, ecological water quality, and urban greening had no significant effect on SRPH and SRMH. Instead, the accessibility of parks and squares, the frequency of usage of parks and squares, and the frequency of sports venue use have a positive impact on SRH. This outcome indicates that people in the central areas of the city are less concerned with the quality of air, water, and green but pay more attention to the actual convenience of health facilities for daily use. This result may be due to the fact that the environmental quality in the downtown area of Chengdu has been able to fulfill the demands of the majority of inhabitants. Therefore, enhancing the quality and accessibility of parks and squares is more helpful to SRH than merely improving urban greening and water quality.

Our research further confirms a significant correlation between the neighborhood’s social environment and SRMH, with a pleasant social environment having a beneficial influence on SRMH. There is evidence that positive neighborhood social environment features can help mitigate the negative impacts of chronic disease and lessen the influence of disease on poor SRH [53]. Excellent social participation and neighborhood interactions are also associated with better SRH and higher rates of physical activity [29,54]. Positive changes in neighborhood population composition (neighbor’s education level, income, and employment rate) are also related to the improvement of healthy behaviors. Moreover, living in a community with improving characteristics is also associated with improvements in individual healthy lifestyle factors, which interact in a complicated way with the community environment [55,56]. Gomez et al. found no correlation between community safety perception and SRH, whereas, in our model, contentment with the urban security environment is strongly linked to SRMH [31]. According to studies, a strong connection exists between neighborhood security and mental health [57]. People who perceive their neighborhood as insecure are likely to reduce social interactions and appropriate health behaviors, a situation which, in turn, can lead to adverse physical and mental health outcomes [58].

The relevance of social environment evaluation for SRH is highlighted in this study. Although no significant correlation occurs between SRPH and neighborhood environmental evaluation in our results, the former is only associated with accessibility and frequency of health service facilities. However, previous studies have shown that a metropolitan physical environment contributes to the improvement of SRPH, which, in turn, affects SRMH [59]. In general, people pay more attention to the physical environment in the construction of neighborhoods and communities, and the social environment is usually neglected. Thus, more attention should be paid to the social environment of the neighborhood, and both the physical and social environments of the neighborhood should be improved together in the neighborhood’s construction.

#### 4.2.2. Frequency of Use and Accessibility of Health Service Facilities Play an Important Role in the Influencing Factors of SRH

High walkability in the community (including park accessibility, park quality, and accessibility of sports facilities) is related to health-improving behaviors, which are positively related to SRH [60,61]. According to our findings, high perceived accessibility of park squares is associated with positive SRMH, and high accessibility of sports venues was associated with positive SRPH. Parks and squares are essential community meeting spaces that can provide residents with opportunities to approach others, exercise, and engage in active and passive social interaction, thereby improving the health level of self-evaluation [59]. Sports zones have little correlation with SRH, and their perceived accessibility is not always related to actual use.

Lan Wang et al. proposed that the farther the distance from the residence to the medical service station, the less the proportion of residents who perceived their health status as good or better [62], which is consistent with the partial results of this study: the higher the perceived accessibility of general hospitals, the higher the SRPH level. In addition, the results showed that the frequency of use of general hospitals was negatively correlated with SRPH, which may be that residents with poorer physical health status have a more significant demand for general hospitals and are more likely to use general hospitals more frequently. Interestingly, our research confirmed that the perceived accessibility of community hospitals is negatively correlated with SRMH. One possible explanation might be that most residents feel that community health services have more negative health impacts (such as infectious illnesses and crowding) and that the higher the accessibility, the larger the negative health effects. However, the number and accessibility of general hospitals are much lower than those of community hospitals, and the adverse psychological effects brought by general hospitals are minimal.

According to our findings, the frequency with which people visit parks, squares, and sports zones has distinct effects on SRPH and SRMH. The use of parks, squares, and sports zones on a regular basis is more favorable for good SRMH and has a greater influence on enhancing SRPH. Parks and squares have long been proven to effectively relieve the pressure on users, reduce the occurrence of depression, restore residents’ vitality, and enhance peace and stability [63,64]. At the same time, those features have the potential to promote certain habits by affecting people’s perceptions and therefore have an impact on their health. Of course, other studies have indicated that SRH is not significantly related to the use of green park space but is related to the vegetation type of land cover [33]. This result reminds us that while considering the accessibility of park squares and sports facilities, we should also pay attention to the diversity of its natural environment and the matching of facilities. In the context of rapid urban expansion, the city has not allocated much land to parks, squares, and sports venues. Therefore, maintaining and beautifying the parks and sports facilities in the community rather than rebuilding them on vacant land is particularly important [30].

In general, interactions may occur among neighborhood facilities, physical environment, social environment, and health and well-being, and we should not focus on only one of these aspects. City planning is, therefore, an essential element of a multi-level, multisector response to face major global health challenges [65]. In addition, the factors that affect SRMH and SRPH are distinct, and a high correlation occurs between mental health and physical health. While paying attention to physical health, we should also focus on the mental health of residents so as to meet the overall health needs of residents and reduce health disparities between neighborhoods.

### 4.3. Actual Accessibility of Health Services near Settlements Should Be Considered More Seriously

Our results suggest that both objective and subjective measures of residents’ perceptions of health services near their settlements may independently influence their own health and well-being. The actual accessibility of facilities has a greater impact on SRPH than perceived accessibility. Clearly, the actual accessibility of sports venues and sports zones is significantly associated with SRPH, whereas the perceived accessibility is not. We know that residents’ perceptions of the physical environment are inextricably linked to their social environments [66]. The deviation between residents’ perception of facilities and the actual situation could be due to the current social environment’s lack of attention to sports facilities and given residents’ preference for using park squares in daily life. Furthermore, both actual and perceived accessibility of health services had a smaller effect on SRMH.

### 4.4. Strengths and Limitations

One of the strengths of this study is that we have made a more detailed treatment of SRH in the study, divided it into SRPH and SRMH, and compared the influencing factors of the two. In addition, we used two different data collection methods.

However, this study has certain limitations, such as the lack of an evaluation of lifestyle factors, including smoking, alcohol misuse, and food choices, as well as factors such as slow walking or major illness. There may be a correlation between mental health and physical health, and this study ignores the interaction between the two. An index can also be established to display overall health (physical health + mental health) and explore the relationship between overall health and the neighborhood environment in future research. At the same time, research should include individual medical history, lifestyle factors, and psychosocial activities that may interact with the environment and health. Furthermore, the results of this study show that significant differences occurred between residents’ perceived accessibility and actual accessibility of facilities on SRH, which also indicates the limitations of this study: the answers of respondents may not reflect the reality. The respondents in this work may represent a more active group of residents whose views are likely to produce recall bias, thereby leading to a more favorable overall view of the surrounding environment.

## 5. Conclusions

Health service facilities that include physical activity facilities and medical and health facilities are critical to the physical and mental health of residents. We analyzed the influencing factors of SRPH and SRMH of residents in the central city of Chengdu using questionnaire data and POI data from field research and employed mathematical, statistical, and GIS tools to compare the influencing differences between the perceived and actual accessibility of health service facilities. In our study, the influencing factors explored are no longer limited to the traditional factors contained in the neighborhood’s social environment and physical environment but rather include factors such as frequency of use, evaluation with, and accessibility of health service facilities. These additional factors were significantly associated with SRPH and SRMH, mainly in the parks, squares, and some medical facilities. This finding suggests that urban planners and managers must pay attention to the development and management of health service facilities, particularly in terms of maintenance design and accessibility, so that each resident can obtain a more accurate perception of and a higher willingness to use them. At the same time, the social environment and the physical environment of the neighborhood interact with each other, and both work together to improve the physical and mental health of the residents and require a reasonable combination and design of the social and physical environment to promote the residents’ access to good environmental perception and the improvement of their physical and mental health.

## Figures and Tables

**Table 1:** Description of variables.

	*n*	WEIGHTED (%)	Average SRPH Scores	Average SRMH Scores
Gender				
Male	288	48.7	3.98	3.80
Female	303	51.3	3.86	3.71
Age (years)				
The young	377	63.8	3.94	3.69
The middle-aged	192	32.5	3.88	3.83
The old	22	3.7	3.91	4.14
Education				
High school degree or below	140	23.7	3.84	3.80
Junior college	120	20.3	3.98	3.66
Bachelor degree or above	331	56.0	3.93	3.77
Permanent job				
Yes	381	64.5	3.92	3.77
No	210	35.5	3.92	3.73
Marriage				
Single	299	50.6	3.95	3.67
Married	292	49.4	3.89	3.84
Annual income (RMB)				
<50,000	219	37.0	3.89	3.77
50,000–100,000	206	34.9	3.93	3.66
>100,000	166	28.1	3.96	3.85
Type of dwelling				
Low-end	89	15.1	3.89	3.65
Mid-range	407	68.8	3.92	3.74
High-end	35	5.9	3.83	4.03
Unit/dormitory	60	10.2	4.02	3.83

Note: Variables (*n* = 591).

**Table 2:** Descriptive statistics of residents’ evaluation scoring results.

	Neighborhood Physical Environment Evaluation						Neighborhood Social Environment Evaluation		
	Air Quality	Ecological Water Quality	Urban Greening	Garbage Disposal	Sports Facilities	Medical and Health Facilities	Community Neighborhood Relations	Social Environment	Urban Public Security
Mean	3.45	3.35	3.70	3.37	3.23	3.27	3.62	3.64	3.80
SD	0.92	0.93	0.82	0.97	1.22	1.17	0.83	0.79	0.82

Note: SD = Standard deviation.

**Table 3:** Chi-square tests of various health service facilities and SRH.

Various Health Service Facilities	Actual Facility Accessibility				Perceived Facility Accessibility			
	Physical Health		Mental Health		Physical Health		Mental Health	
	χ2	*p*	χ2	*p*	χ2	*p*	χ2	*p*
Parks and Squares	19.23	0.014 **	5.24	0.731	21.67	0.017 **	15.43	0.117
Sports Venues	19.19	0.014 **	11.10	0.196	6.09	0.807	6.07	0.809
Sports Zones	15.48	0.051 *	6.12	0.633	2.54	0.990	9.70	0.467
General Hospitals	14.27	0.075 *	4.75	0.784	24.74	0.006 **	24.17	0.007 **
Specialised Hospitals	7.48	0.679	15.25	0.123	14.49	0.152	9.03	0.529
Community-Hospitals	7.72	0.461	4.21	0.837	17.20	0.070 *	5.693	0.840
Clinics	15.12	0.057 *	7.57	0.476	6.48	0.773	14.06	0.170
Pharmacies	12.59	0.247	5.63	0.845	3.57	0.965	13.66	0.189

Note: ** and * respectively represent *p* < 0.05, and *p* < 0.10.

## Data Availability

The raw/processed data required to reproduce these findings cannot be shared at this time as the data also forms part of an ongoing study.

## Supplementary Materials

The following supporting information can be downloaded at: https://www.mdpi.com/article/10.3390/ijerph19084501/s1, Table S1: SRH scale.
