# Supplementary material for: The Effects of Urban Neighborhood Environmental Evaluation and Health Service Facilities on Residents’ Self-Rated Physical and Mental Health: A Comparative and Empirical Survey
Source: Int J Environ Res Public Health. 2022 Apr 8;19(8):4501. doi: 10.3390/ijerph19084501 (PMC9027638; doi:10.3390/ijerph19084501)
Supplement: Supplementary file 1 [file ijerph-19-04501-s001.zip › ijerph-1578257-supplementary.pdf]

Table S1. SRH scale

**SRMH Scale: Warwick-Edinburgh Positive Mental Health Scale (WEMWBS)**			
1	I've been feeling optimistic about the future	8	I've been feeling good about myself
2	I've been feeling useful	9	I've been feeling close to other people
3	I've been feeling relaxed	10	I've been feeling confident
4	I've been feeling interested in other people	11	I've been able to make up my own mind bout things
5	I've had energy to spare	12	I've been feeling loved
6	I've been dealing with problems well	13	I've been interested in new things
7	I've been thinking clearly	14	I've been feeling cheerful
1= Not Often, 2= Rarely, 3= Sometimes, 4= Often, 5= Always			
**SRPH**			
1	Overall, how do you rate your health compared to your peers?		
1 = Poor, 2 = Average, 3 = Good, 4 = Very Good, 5 = Excellent			
